# Mania Associated With Herbal Medicines, Other Than Cannabis: A Systematic Review and Quality Assessment of Case Reports

**DOI:** 10.3389/fpsyt.2018.00280

**Published:** 2018-07-06

**Authors:** Emmanuelle Bostock, Kenneth Kirkby, Michael Garry, Bruce Taylor, Jason A. Hawrelak

**Affiliations:** ^1^Psychology, University of Tasmania, Hobart, TAS, Australia; ^2^Psychiatry, University of Tasmania, Hobart, TAS, Australia; ^3^Neurology, Menzies Institute for Medical Research, Hobart, TAS, Australia; ^4^College of Health and Medicine, University of Tasmania, Hobart, TAS, Australia; ^5^Australian Research Centre for Complementary and Integrative Medicine, University of Technology Sydney, Ultimo, NSW, Australia

**Keywords:** herbal medicine, case report, bipolar disorder, mania, phytotherapy

## Abstract

**Background:** DSM-5 introduced the diagnostic category of substance/medication-induced bipolar and related disorder. This systematic review examines published reports linking mania with the consumption of herbal medicines (HM), excluding cannabis. Putative pathophysiological mechanisms that may account for the reported HM being associated with mania are discussed.

**Methods:** A systematic search of EMBASE, CINAHL, Health Source, PsychINFO, and PubMed. The quality of case reports meeting inclusion criteria was assessed using the modified Quality Assessment Scale by Agbabiaka.

**Results:** Nineteen single and seven multiple-case reports met inclusion criteria. These yielded a study sample of 35 case reports, 28 of herbal medicine associated mania, 5 of hypomania, and two mixed states, in 17 females [age in years M(SD) = 43.1(13.2)] and 18 males [40.7(18.1)]. A total of 11 herbal medicines were implicated. Case reports by herbal medicine (number of reports) comprised: St John's wort (*Hypericum perforatum)* (14); Ginseng (*Panax ginseng*) (5); brindleberry (*Garcinia cambogia*) (4); ma-huang (*Ephedra sinica*) (3); “herbal slimming pills” (2); Herbalife products (2); Hydroxycut (1); horny goat weed (*Epimedium grandiflorum*) (1); “herbal body tonic” (1); celery root (*Apium graveolans*) (1), and a “herbal mixture” (1). All case reports were associated with use rather than withdrawal of herbal medicines. Only one case report was rated for probability of association using a standardized algorithm. Laboratory assays to confirm composition of the herbal preparation were reported in only one article describing two cases and indicating admixture of a likely causal pharmaceutical in the herbal preparation.

**Conclusions:** Causal attributions are problematic given the limited number of reports, antidepressant co-prescribing in 7 cases, insufficient data regarding pattern and type of herbal medicine use, and lack of a reference frequency for spontaneous mania.The quality assessment scores across the 26 papers (35 case reports) were as follows: low quality (0), lower-medium quality (9), upper-medium quality (10) and high quality (7). Putative pathophysiological mechanisms were postulated for nine of the 11 herbal medicines and centered on HPA-axis activation and increased monoamine activity. Systematic study of the association between herbal medicines and the course of bipolar disorder may contribute to defining targets for pathophysiological research.

## Introduction

DSM-5 introduced the diagnostic category of “substance/medication-induced bipolar and related disorder.” This diagnosis requires a temporal association between occurrence of mania and the use or withdrawal of substances or medications. The precipitating agents may include intoxicating drugs such as cannabis or amphetamines, prescribed medications for mood disorders such as antidepressants, prescribed medications for other illnesses such as steroids, and herbal medicines (HM). DSM-5 sets a less restrictive standard for the diagnosis of substance/medication-induced bipolar and related disorder than for mania. Criterion A for mania, as required for a diagnosis of bipolar disorder, is “A distinct period of abnormally and persistently elevated, expansive, or irritable mood and abnormally and persistently increased activity or energy, lasting at least 1 week and present most of the day, nearly every day (or any duration if hospitalization is necessary)” (1). This compares with criterion A for substance/medication-induced bipolar and related disorder “a prominent and persistant disturbance in mood that predominates in the clinical picture and is characterized by elevated, expansive or irritable mood.”

The concept of substance/medication-induced mania antedates the DSM-5 classification, for example it was denoted as bipolar disorder-III in Akiskal's classification of bipolar spectrum disorders (2). Mania associated with the antidepressant medication imipramine was reported by Ball and Kiloh (3) and mania has also been associated with lithium withdrawal (4). The first reported association of mania with a HM was in 1984 by Price et al. who explored the relation of yohimbine, an α-2 adrenergic receptor antagonist, to mania under experimental conditions (5). A number of substances have beneficial effects in bipolar disorder and are prescribed medications in routine treatment of mania for example lithium, sodium valproate and atypical antipsychotics, and in bipolar depression for example lithium, lamotrigine, atypical antipsychotics and selective serotonin reuptake inhibitors (6). Their precise mechanism of action is not fully understood, reflecting a lack of understanding of the pathophysiology of mania and depression. The study of substance/medication-induced bipolar and related disorder, notwithstanding limitations of causal attributions, may further our understanding of brain mechanisms relevant to the occurrence and course of bipolar disorder. This is particularly so for mania which has a strikingly unique set of clinical features, is often of abrupt onset and has a relatively short duration with a median of 13 weeks (7).

Many conventional drugs originate from plant sources (8). HM or phytotherapy refers to the use of plant-based medicinal preparations, a subset of complementary and alternative medicines (CAM). In the United States, HM are regulated as food products and therefore are not subject to the phases of clinical testing that pharmaceuticals must undergo prior to market release. Manufacturing standards are in keeping with those applicable to other foods (9), the strength and composition of HM may therefore vary widely.

According to survey data, the use of CAM is prevalent and increasing throughout many Western countries (10–12). CAM usage is common in persons with psychiatric illness. In a survey of CAM usage among psychiatric inpatients (*n* = 82) it was found that 63% used at least one CAM modality within the previous 12 months, including 44% who used HM (13). This may be attributed in part to factors such as side effects of conventional medicines, ready access without prescription, a belief that HM cause no harm, and in the case of bipolar illness, traits such as novelty seeking in mania or hypomania (14, 15). In a survey of 826 new patients presenting at a CAM clinic, 578 (70%) had a mental disorder and reported lower quality of life and greater levels of stress than those without a mental disorder. Among patients with a mental disorder, the major reasons for choosing complementary therapies were personal preference, interest, or beliefs in complementary therapies (44.3%) including as a treatment of last resort (30.7%) (16).

In a cross-sectional survey of 100 older (>55 years) inpatients and outpatients with bipolar disorder (*n* = 50) or major depression (*n* = 50), the use of herbal and nutritional compounds (HNC) was examined to determine several factors including, knowledge of products, perceived efficacy and safety, patterns of use and discussion of use with health care providers. Approximately 30% of respondents reported using oral HNC, 40% thought that they were Food and Drug Administration regulated and 14–20% preferred to take HNC to psychotropic medications (17).

A review of CAM therapies in the treatment of bipolar disorder, noted that few rigorous clinical studies have been conducted in this patient population (14). The herbal preparation Free and Easy Wanderer Plus has been examined as an adjunct to carbamazepine (CBZ) in a double-blinded, randomized placebo-controlled trial in patients with bipolar disorder in manic and depressive phases (18). When compared to CBZ monotherapy, at week 4 and 8 of the trial, the HM combined with CBZ resulted in significant improvement in depression but not mania.

Although cannabis has largely been seen as an illicit drug, it is now entering into conventional medicine under the rubric of “medicinal cannabis.” Cannabis has been extensively researched in relation to its acute and chronic effects in psychosis. Discussion of the role of cannabis in bipolar disorder is beyond the scope of this article but is summarized in a systematic review and meta-analysis by Gibbs et al. (19). This reported that, on balance, in pre-existing bipolar disorder, cannabis may worsen the occurrence of manic symptoms and may also act as a causal risk factor in the incidence of manic symptoms.

This paper presents a systematic review of single and multiple-case reports of mania associated with herbal medicines other than cannabis. A more comprehensive understanding of what precipitates mania in vulnerable individuals may potentially lead to new understandings of the illness and the substrates that are implicated in bipolar disorders.

## Method

This review of reports of herbal medicine-associated mania was conducted in accordance with the Preferred Reporting Items for Systematic Reviews and Meta-Analyses (PRISMA) guidelines (20).

*Inclusion Criteria:* (a) published between 1980–2017 (b) in a peer-reviewed journal (c) included adult participants (> 18 years) (d) published in the English language. *Exclusion criteria:* (a) psychosis in the absence of manic features (b) secondary manias due to infection, neoplasm, epilepsy, and metabolic disturbances (c) systematic reviews (d) relating to cannabis.

### Identification of studies

In the first week of September 2017, a search of the electronic databases EMBASE, CINAHL, Health Source, PsychINFO and PubMed was conducted to find published associations between HM and mania. The search was commenced by identifying in each database the controlled vocabulary terms/ subject terms related to herbal medicines (group 1) and bipolar disorder (group 2). All subject terms were exploded. Subject terms in the respective databases for group 1 were herbal medicine (PubMed); herbal medicine and medicinal herbs and plants (EMBASE and PsychInfo); medicine and herbal medicine (CINAHL and Health Source). Additionally, 182 free text terms that related to herbal medicines, including botanical names, (see Appendix 1) were combined with “OR.” Subject terms for group 2 were bipolar disorder, cyclothymic disorder (PubMed); bipolar disorder, cyclothymia (EMBASE and PsychInfo); and bipolar disorder (CINAHL and Health Source). The free text terms bipolar disorder, mania, cyclothymia, manic-depressive psychosis, manic state, bipolar depression and manic disorder were searched for and combined with “OR.” Finally, group 1 and group 2 were combined with “AND.”

A purpose-built coding sheet was used to assess articles against the inclusion criteria. To assess accuracy of initial screening KK and EB separately rated 20 titles and abstracts, randomly selected from the records screened using the RANDBETWEEN function in Microsoft Excel version 15.23.2. Inter-rater agreement on exclusion/inclusion was 100%.

### Quality assessment of case reports

Authors EB and KK individually assessed all included case reports for quality, according to the following nine criteria: classification on Quality Assessment Scale by Agbabiaka; use of a validated instrument to assess for causality (Naranjo or WHO-UMC score); botanical name of herbal medicine stated; herbal material assessed for authentication; herbal material assessed for adulteration; characteristics of herbal medicine detailed (e.g., plant part, extract type); product brand name and manufacturer detailed; batch numbers; herbal dosage specified. The case report Quality Assessment Scale by Agbabiaka (21) as modified by Hung, Hillier and Ernst (22) has 21 questions rated as “yes,” “unclear,” and “no,” each item scored between 0 and 2 points for a total score out of 42 points. Each case report was classified as: low quality (0-14 points), lower medium quality (15–21 points), upper medium quality (22–28 points) or high quality (29–42 points) as recommended by Agbabiaka (21).

After each paper had been assessed EB and KK discussed the results and came to a consensus score. Inter-rater reliability on the Quality Assessment Scale by Agbabiaka was calculated using several indices: raw agreement (number of agreements for individual items divided by number of possible agreements), kappa coefficient and intraclass correlation coefficient (ICC). Raw agreement ranged from 52.4 to 95.2% for individual articles with mean raw agreement 75.6% (95% CI [71.2%, 80.1%]). Separate kappa coefficients were calculated between the two raters for all items on each of the 26 articles. To obtain mean kappa, the 26 kappa coefficients were first transformed using Fisher's transformation to achieve linearity. The mean and 95% CIs were calculated, then back-transformed to original kappa units. Individual kappa coefficients ranged from 0.16 to 0.92. Mean kappa was 0.60 (95% CI [0.50, 0.68]). Applying the criteria of Landis and Koch this represents a moderate-to-substantial level of agreement (23). The ICC between the two raters was calculated from the total score for each article using two-way ANOVA (24). Each item on the scale was rated as 0, 1, or 2. With 21 items the maximum possible score for an article was 42. Rater and article were treated as separate, random effects in the model. For total scores the means were 24.5 (*SD* = 6.2) for EB and 24.7 (*SD* = 5.6) for KK. The inter-article correlation between raters was 0.70 (95% CI [0.42, 0.85]). The ICC was 0.82 (95% CI [0.60, 0.92]) indicating a good-to-excellent level of agreement (25).

## Results

**Figure 1:**
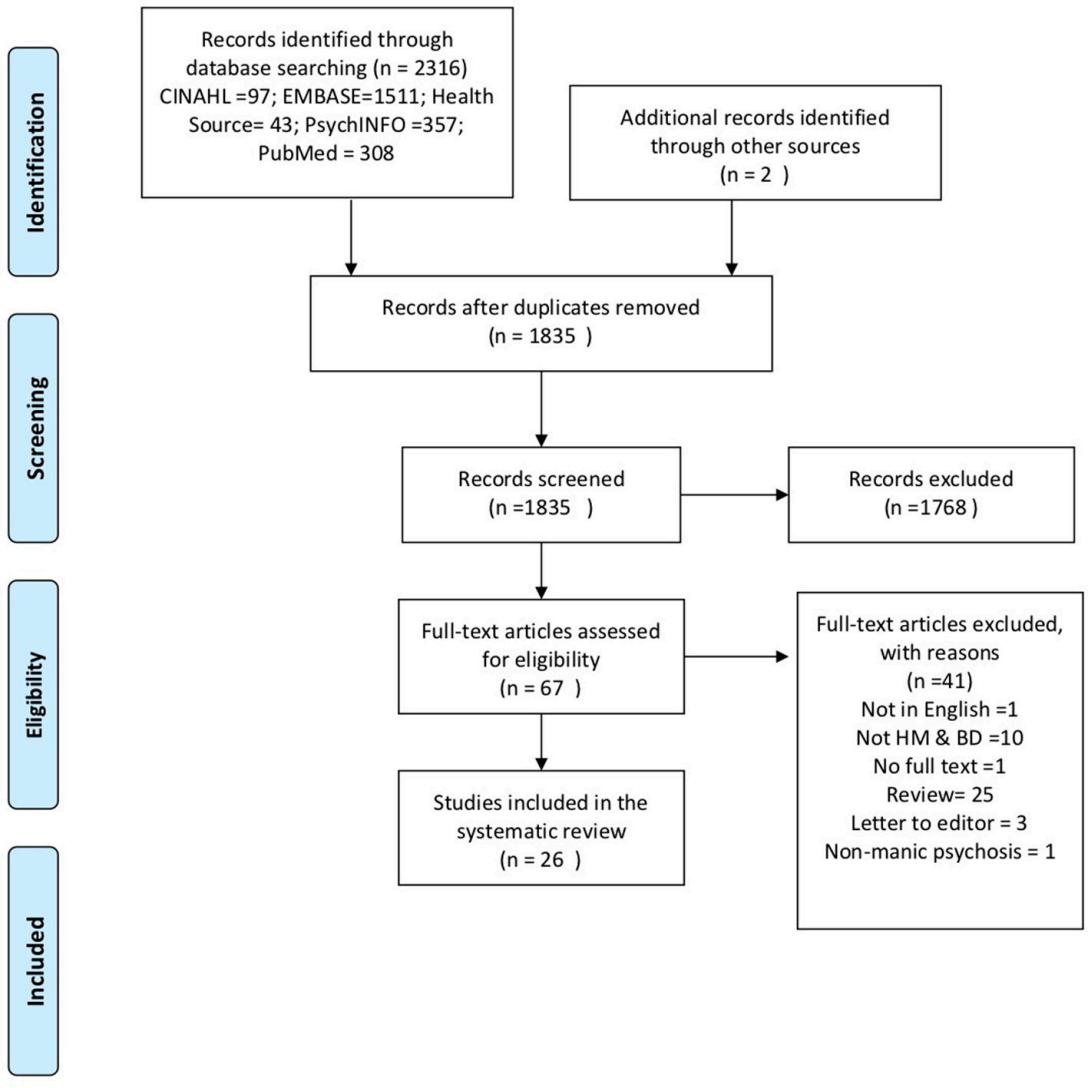
Preferred reporting items for systematic reviews and meta-analysis flow diagram of included studies.

The results of the search strategy are summarized in the PRISMA diagram (Figure 1). There were no randomized clinical trials of HM in mania. The study sample comprised 35 case reports, from 19 single and 7 multiple-case reports, of an association between mania and use of HM. Details of each case report are summarized in Table S1, including clinical and demographic details, the composition of the HM, other medications, and treatment outcomes.

In summary, the reports included 17 females [age in years M(SD) = 43.1(13.2)] and 18 males [40.7(18.1)]. Case reports, grouped by HM (number of reports) comprised: St John's wort (*Hypericum perforatum*) (14); Ginseng (*Panax ginseng*) (5); brindleberry (*Garcinia cambogia*) (4); ma-huang (*Ephedra sinica*) (3); “herbal slimming pills” (2); Herbalife products (2); Hydroxycut (1); horny goat weed (*Epimedium grandiflorum*) (1); “herbal body tonic” (1); celery root (*Apium graveolans*) (1), and “herbal mixture” (1). Fourteen cases were taking concurrent prescription medications, comprising antidepressant (SSRI 3; SNRI 2; tricyclic 1; NDRI 1); lithium (1); antipsychotic (2); atypical antipsychotic (1); anti-epileptic (2); statin (2); beta-blocker (1); NSAID (2); sildenafil (1) and a synthetic glucocorticoid (1). A psychiatric history was noted in 24 of the 35 cases of HM-associated mania, diagnoses included depression (*n* = 11) bipolar disorder type I (BDI) (*n* = 5), bipolar disorder type II (BDII) (*n* = 2), post-traumatic symptoms (*n* = 1), eating disorder (*n* = 2), past suicide attempt (*n* = 1) and substance/medication-induced bipolar and related disorder (*n* = 1). For the whole sample the time to onset of manic symptoms from commencing the HM was between 2 days and 2 years with a median of 4 weeks.

### St John's wort (*hypericum perforatum*)

Fourteen case reports described mania, hypomania and two mixed states associated with St John's wort, in seven females [age in years M(SD) = 49.4(12.4)] and seven males [39.4 (17.8)]. Stated reasons for taking St John's wort were depression (12) (26–34), to improve energy (1) (29) and to relieve symptoms of post-traumatic stress (1) (29). The time of onset of manic symptoms from commencing HM ranged from 3 days to 2 months. The mental status on examination was consistent with mania and two mixed state in the 14 cases. Three cases had a past psychiatric history of bipolar disorder, and eight of unipolar depression of whom four were concurrently taking antidepressants. In eight cases, the dose of the St John's wort preparation was not specified.

### Ginseng (*panax ginseng*)

There were five case reports of *Ginseng*-associated mania in our study sample, two females [46(10) years] and three males [42.7(25.7)]. Stated reasons for taking *Ginseng* were to boost energy (2) (35, 36), fatigue (1) (37), erectile dysfunction (1) (34) and one unknown (1) (38). The time to onset of symptoms between taking *Ginseng* and mania ranged between 10 days and 2 months prior. Of these five cases, two had a prior history of depression, one of substance-induced hypomania, two had no past psychiatric history. The reported range of daily doses of *Ginseng* in the case reports were 500–750 mg of root or 300mg−20 g of extract, compared to a recommended short-term dose range of 0.5–2 g of dry *Ginseng* root, equivalent to 200–600 mg of extract, and long-term dose of 1 g of dry root (39). In two cases, the recommended long-term dose was far exceeded.

### Brindleberry (*garcinia cambogia*)

There were two case reports of mania and one each of manic psychosis and hypomania, involving *Garcinia cambogia*, 2 females [42.5(8.5) years] and 2 males [37.5(12.5)]. The stated reason for taking the HM was weight loss (40, 41) and three patients had a past history of bipolar disorder. In each case, the dose of *Garcinia cambogia* was not specified. The time to onset of manic symptoms from commencing the HM ranged from 2–6 weeks. Two cases were concurrently taking antidepressants and mood stabilizers.

### Ma-huang (*ephedra sinica*)

In three cases, ma-huang (in one case along with chromium picolinate and caffeine) was associated with manic-like symptoms in individuals without a history of bipolar illness, two females [30.5(9.5) years] and one male [45 years]. Stated reasons for taking the HM were weight loss (42, 43) and heightened alertness and to prevent drowsiness (44). Past psychiatric history included hospitalization for alcohol poisoning, and bulimia without purging with a description of manic-like symptoms. The family psychiatric history included possible bipolar disorder and schizophrenia. The time to onset of manic symptoms ranged between 5 days and 2 months. In each case the dose of the HM was unknown, concurrent medication in one case comprised thyroid hormone and recently discontinued antidepressant.

### Herbalife products

Two cases reported mania associated with the use of a Herbalife product for the stated reason of weight loss (45, 46). Both were male [32.5 (6.5)] and neither had a personal or family history of bipolar disorder or were taking prescribed medication. In one case, the patient became manic after taking “large amounts” of the products both as tablets and tea. The time to onset of manic symptoms ranged between 2 and 20 days. In a subsequent published letter regarding the above case reports, by authors affiliated with the Herbalife brand, (47), it was noted that the case reports did not specify the precise product (as opposed to the brand) and/or ingredients taken in these two cases.

### Herbal slimming pills

In a multiple-case report by Chong, two cases of manic-like psychosis were associated with the use of “Herbal slimming pills” (48). An assay of the HM identified that it contained the anti-depressant pharmaceutical sibutramine. The authors attributed the occurrence of mania to this adulterant.

There were five single case reports that related an HM (ingredients see Table S1) to mania, involving Hydroxycut (49), Horny goat weed (*Epimedium grandiflorum*) (50), “herbal body tonic,” prescribed for anger (51), celery root (*Apium graveolans*) for menopausal symptoms (52) and a “herbal mixture” prescribed for fatigue (53).

## Quality assessment of case reports

In addition to the systematic review of HM associated mania, we also assessed the quality of the individual case reports. The results are shown in Table S2.

Applying Hung, Hillier and Ernst's (22) modified version of the Agbabiaka tool (54), the distribution of the quality assessment scores across the 26 papers, covering 35 case reports, was as follows: low quality (0), lower-medium quality (9), upper-medium quality (10) and high quality (7). Only one single case report used a validated instrument to assess causality, the Naranjo scale. The botanical name was listed in 19 cases in 18 papers. Only 1 paper presenting 2 case reports used a laboratory assay to confirm the composition of the HM. The composition of the HM, for example plant part used and extract type, was detailed in only 1 case report. The brand name and manufacturer were stated in 3 case reports. The dosage of the HM was specified in only 18 of the case reports. Of the study sample, 2 case reports of 1 HM were assessed for adulteration. The batch number was provided in none of the case reports. Herbal dosage was detailed fully in 6 and partially in 3 cases.

## Discussion

This review examined case report evidence regarding HM-associated mania. Those included were: St John's wort (*Hypericum perforatum)*; Ginseng (*Panax Ginseng)*; Brindleberry (*Garcinia cambogia)*; ma-huang (*Ephedra sinica*); “herbal slimming pills”; Herbalife products; Hydroxycut; horny goat weed (*Epimedium grandiflorum*); “herbal body tonic”; celery root (*Apium graveolans*), and “herbal mixture.” Where possible the candidate pathophysiological mechanisms are discussed in turn, as are other factors which may have contributed to the onset of mania in the individuals included in the study. There is an inherent difficulty in making attributions regarding the causality of HM on mania as the course of bipolar disorder is unpredictable.

Of the 35 case reports included in this review, 5 were isolated reports of one HM and two cases (both “herbal slimming pills”) were attributed to a contaminant. The remaining 28 cases were accounted for by five HM, all of which were the subject of two or more case reports of mania. Over a publication period of 38 years (1980–2017) this is a small yield of reported concurrence of HM usage and mania. The small numbers and unreliability of discerning and reporting a link between mania and HM preclude any definitive statement as to whether any association is causal or coincidental.

With respect to St John's wort, there is a high specificity of the stated reason for taking the HM to be for treatment of depression (11 of 14 St John's wort cases compared to 0 of 21 other HM). There was also greater morbidity in the psychiatric history of the St John's wort cases (13 of 14 cases compared to 11 of 21 for other HM), in the family history of mood disorder (6 of 14 compared to 2 of 21) and concurrent antidepressant prescribing was more common (4 of 14 compared to 4 of 21). Competing explanations for these patterns include an increased diathesis toward bipolar disorder, fluctuations of established affective illness, antidepressant-associated mania and HM-associated mania. These factors are not mutually exclusive, for instance according to Craddock and Sklar, a family history of bipolar disorder is an important clinical predictor of a likely bipolar course in a patient who presents with one or more episodes of depression even before their first personal episode of mood elevation (55).

The mechanism by which St John's wort may alter susceptibility to mania is not well understood. As with antidepressants, it is difficult to distinguish spontaneous episodes of mania from St John's wort-associated switching (56). Angst et al. analyzed the time course and risk factors for a diagnostic change from major depression to bipolar disorders over an average of 20 years from onset. Diagnostic change from depression to bipolar type I occurred in approximately 1% and bipolar type II 0.5% of patients per year (57). In patients with major depressive disorder treated with antidepressants, it has been found that antidepressant-associated mania or hypomania occurs at an average frequency of 3.42% of cases per year, but it is unclear to what extent switching represents undiagnosed bipolar disorder or a direct pharmacological effect of antidepressants (58).

Depression is one of the most commonly cited reasons for using CAM (59). The prevalence of depression in the United States has been reported to have increased between 2005 and 2015 (60). For many patients with depression, HM which are in many countries, predominantly available over-the-counter, may be an attractive alternative to conventional medicines. There is a substantial evidence base from randomized controlled trials supporting the use of St John's wort in mild-to-moderate depression. In a systematic review and meta-analysis comprising 23 randomized trials of St John's wort in outpatients with mild-to-moderate depression (*N* = 1757), it was found that *Hypericum perforatum* extracts were significantly more effective than placebo (61).

St John's wort has a variety of actions that may contribute to its therapeutic effects. *In vitro*, it acts on neurotransmitter regulation, including beta adrenergic and glutamate receptors, and ion channel conductance. Hypericin (an active constituent of St John's wort) inhibits serotonin reuptake, and 5-HT_1A_ and 5-HT_1B_ receptor changes are associated with prolonged use (62). According to Fahmi et al, in animals, hypericum is effective in three major biochemical systems relevant for antidepressant activity including inhibition of synaptic reuptake of serotonin, noradrenaline and dopamine (26). In the human case reported by Barbenel of concurrent prescribing of St John's wort and sertraline in a patient following surgery for crypto-orchidism, alteration of testosterone and gonadotrophin levels and interactions of antidepressant and St John's wort were further considerations.

With five case reports of *Ginseng* associated mania it was the second most commonly reported HM after St John's wort. Based on the belief that it is a panacea and promotes longevity, *Ginseng* root has been used for over 2000 years (63). There are a number of plants that share the common name *Ginseng* however only three of these are from the genus Panax (*Panax ginseng, P. notoginseng* and *P. quinquefolius*). Other “ginsengs” include Siberian (*Eleutherococcus senticosus*), Indian (*Withania somnifera*), and Brazilian (*Pfaffia paniculata*) (64, 65). The most important constituents of Panax ginseng are the ginsenosides, of which 15 different types have been identified (35).

In a systematic review of RCTs examining the efficacy of *Panax ginseng* root extracts for a number of indications, it was concluded that there is contradictory evidence that *Ginseng* improves physical performance and immunological measures. It may have beneficial effects on psychomotor performance and cognitive behavior. No trial has confirmed the alleged age-delaying properties of *Ginseng*. Results suggesting a reduction of blood glucose levels in type-II diabetic patients require further investigation (64, 65). With *Panax* g*inseng*, two mechanisms of action in depression have been advocated, firstly, an activating effect of ginsenosides on the HPA-axis resulting in elevated corticotropin and corticosteroid levels (33). Secondly, monoamine signaling could also be affected by ginsenosides (66, 67).

Thirteen of 35 case reports in our study sample involved adverse psychiatric effects of weight loss products including brindleberry (*Garcinia cambogia*), ma-huang (*Ephedra sinica*), “herbal slimming pills,” Herbalife products and Hydroxycut. The social stigma of obesity, a desire to lose weight without making drastic lifestyle changes, and frustration at previous failed attempts are commonly reported reasons for using dietary supplements which are readily available and advertised as being “natural” and safe (68).

In one 12-week randomized placebo-controlled trial, *Garcinia cambogia* failed to produce significant weight loss and fat loss beyond that observed with a placebo (69). In contrast, another double-blind placebo RCT found that *Garcinia cambogia* reduced abdominal fat accumulation in participants (70). Other human research has confirmed the potential of *Garcinia cambogia*/HCA in stimulating fat oxidation, increasing serotonin release in brain cortex and normalizing lipid profiles (71). The main active ingredient of *Garcinia cambogia* is hydrocitric acid which has serotonergic effects and has been implicated in serotonin syndrome (38). Hydrocitric acid is the putative mediator of this HM weight loss effect; it is thought to promote the release and synaptic availability of serotonin thus influencing appetite (41). The effects of Hydroxycut were attributed to the inclusion of *Garcinia cambogia* in the preparation (49).

Partin and Pushkin, who reported a case of hypomania associated with horny goat weed (*Epimedium grandiflorum*) proposed that this may have been due to the addition of other unidentified herbs and pharmaceuticals (50). However, these were not explicitly tested for.

Ma-huang (*Ephedra sinica*) is native to China and Mongolia and contains sympathomimetic compounds known as *Ephedra* alkaloids. Traditionally used to treat asthma and hay fever symptoms, more recently it has been combined with caffeine or botanical sources of caffeine (for example *Guarana*) for weight loss purposes (68). In a 6–month RCT of herbal *Ephedra*/ caffeine for weight loss, it was found that 90/192 mg/day of herbal *ephedra*/caffeine promoted weight and body fat reduction (72). In another randomized double-blind trial of a herbal supplement containing ma-huang-guarana for weight loss, it was found that the active treatment produced significant effects (73). Ma-huang contains variable amounts of ephedrine congeners which enhance norepinephrine release in central noradrenergic neurons. Ephedrine also has direct agonist activity at alpha and beta-adrenergic receptors (44).

Sibutramine is an appetite-suppressing agent that is a norepinephrine and serotonin reuptake inhibitor, initially developed as an antidepressant (48) its use has been associated with mania (74, 75) and hypomania (76). In a recent analytical study of 447 weight loss products, 119 were found to be adulterated with one or more weight loss compounds including sibutramine, its metabolites benzyl sibutramine and desmethyl sibutramine; phenolphthalein; bisacodyl; furosemide; liothyronine (T3); and thyroxine (T4) (77). This demonstrates the importance of having regulatory bodies oversee CAM manufacturing practices and to regularly assess certain classes of CAM products for adulterants (such as weight loss products). These incidences of mania associated with HM weight-loss products highlight the fact that they may be considered safe and harmless by consumers when they have the propensity to trigger adverse events in vulnerable individuals (78).

Khalid et al. reported a case of mania associated with celery root (*Apium graveolans*), St John's wort (*Hypericum Perforatum)* and venlafaxine. Mania ensued shortly after the ingestion of celery root, which belongs to a group of plants classified as the umbelliferous family, which contain phytoestrogens that are structurally similar to estrogen. In this case the patient developed elevated serum venlafaxine levels after taking celery root for menopausal symptoms, suggesting pharmacokinetic potentiation of the venlafaxine level by the celery root as a likely mechanism of induction of mania (52).

Thirteen of the patients were concurrently taking conventional medicines (as shown in Table S1) as well as HM which may have led to herb-drug interactions resulting in mania. This might result from alterations of absorption, distribution, metabolism or elimination of a conventional drug by a herbal product, that is pharmacokinetic effects (79). Alternatively, there may be synergistic effects of a HM and a conventional medicine reflecting common mechanisms of action such as neurotransmitter regulation. Drug dosage is one important factor in such herb-drug interactions as well as in interactions with underlying biological diatheses (80). In approximately one third of the cases reviewed the dosage of the HM was unknown. This limits the ability to make causal inferences regarding the dose of the HM associated with a manic switch in vulnerable individuals.

For the whole sample the time to onset of manic symptoms from commencing the HM (treatment-emergence interval) ranged between 2 days and 2 years with a median of 4 weeks. None of the HM reported associated with mania diverged notably from this median time to onset of manic symptoms. A treatment-emergence interval of 8–12 weeks is deemed to implicate causality; however, a much shorter interval may be necessary to definitively link cause and effect (81). The manic episodes reported in the cases were mostly treated with conventional anti-manic agents, only two reports indicate the outcome of cessation of the HM alone on the course of mania, one indicating remission in 2 days, the other no improvement following a switch into depression. Thus, no general statement can be made with respect to mania resolution on ceasing the implicated HM.

In addition to the systematic review of HM-associated mania, we also assessed the quality of reporting (Table S2) on the modified Quality Assessment Scale by Agbabiaka, in each of the 26 published case report papers. The case report quality assessment score ratings were low quality (0), lower-medium quality (9), upper-medium quality (10) and high quality (7). There are two algorithm-based rating instruments to assign the probability that an adverse event (in this case mania or hypomania) is related to a given exogenous substance, the Naranjo and the WHO-UMC. Of the cases reviewed only one used the Naranjo scale, none used the WHO-UMC. One study compared the two rating scales and found that the WHO-UMC method was more simple and less time consuming compared to the Naranjo probability scale (82). On the 7 additional quality criteria, the quality of reporting was satisfactory only for the inclusion of botanical name of HM (26 of 35 cases) and to a lesser extent HM dosage (18 of 35 cases). On the remaining 5 criteria less than 10% of papers were compliant. It is noted that many of the reports were published before the advent of defined quality criteria ratings, for example 19 of the 26 papers preceded the publication of the modified Quality Assessment Scale by Agbabiaka in 2008. Future published case reports of adverse events should adhere to such criteria in order to improve their overall quality and inferences which may be made from these articles.

The current review of case reports is subject to a number of limitations. Substance/medication-induced bipolar and related disorder may reflect a switch in a previously unexpressed bipolar diathesis. It is possible that the reports in the scientific literature are subject to a confirmation bias in that clinicians are looking for links between mania and recent use of HM. There is also a possible effect of researcher/ publication bias, which has led to the publication of the included studies. Ethically, it is difficult to replicate studies which suggest a potentially harmful effect of various HM.

The compilation of case histories presents a different sample to that commonly seen in a randomized controlled trial setting where fixed inclusion and exclusion criteria apply. This sample of putative HM induced mania includes a number of patients (*n* = 7) with a stated past history of a diagnosis of BD and a further 7 patients without a BD diagnosis taking antidepressants. This increases the likelihood that the observed manic episode was attributable to extant bipolar disorder or antidepressant induced switching rather than the HM *per se*, although interactions of these variables cannot be excluded.

Despite these limitations, however, there are a number of strengths associated with the inclusion of case reports including that the patients, episodes of mania, time course of symptoms and aetiological factors are described in detail, as reflected in Table S1. The authors are generally circumspect in their judgements regarding causality, documenting an observed association, summarizing knowledge regarding possible mechanisms of action, and allowing for the multiplicity of explanations that attend a disorder of unclear etiology and pathogenesis and with a typically fluctuating course.

Concepts of exogenous and endogenous causation of psychoses have been debated over the past century. In 1910, Bonhoeffer proposed that the brain only manifested a few stereotyped mental reactions, whether from exogenous or endogenous origins. He recognized delirium in particular as a presentation that could follow diverse exogenous causes. The typical features in delerium of clouding of consciousness and disorientation form the rationale for categorizing it separately to the psychoses (83). Similarly, in the concept of unitary psychosis, as elaborated by Conrad in 1959, there is no fixed relationship between symptom picture and exogenous factors, the latter potentially triggering a range of symptom pictures (84). Relating these propositions to the current study on case reports of mania associated with herbal medicines, the results are inconclusive because it is restricted to one diagnostic category, mania, and one class of aetiological factors, herbal medicines. Comparisons of reports of herbal medicines associated with the onset of a variety of mental disorders would yield information as to whether the relationship indicates specific aetiologies of a disease (e.g., mania) or a broader relationship whereby a diversity of diagnostic categories (e.g., mania, psychotic depression and schizophrenia) are attributable to a common aetiological factor, the unitarian view.

In summary, the reported co-occurrence of HM usage and mania, whilst inconclusive, provides a plausible signal as to brain mechanisms relevant to the pathogenesis of mania. With the increasing storage of health information in electronic health records and evolving techniques of data mining there are prospects for the widespread application of case report level information to the elucidation of associations and causal links between usage of HM and the occurrence and varied course of mental disorders including BD.

## Author contributions

EB developed the concept of the article from which she received supervision and expert advice in the area of psychiatry from KK, statistics from MG, neurology from BT and complementary medicines from JH.

### Conflict of interest statement

The authors declare that the research was conducted in the absence of any commercial or financial relationships that could be construed as a potential conflict of interest.

## Appendix

**Table:**

	**Search terms**
1	Achillea
2	Adaptogen
3	Albizia
4	Anemone
5	Angelica
6	Asafoetida
7	Ashwagandha
8	Astragalus
9	Atropa
10	Avena
11	Bacopa
12	Ballota
13	Banxia-houpo-tang
14	Betony
15	Bitter orange
16	Borage
17	Borago
18	Botanicals
19	Californian poppy
20	Camellia
21	Cannabis
22	Catmint
23	Catnip
24	Centella
25	Chamomile
26	Chinese medicine
27	Citrus
28	Clove
29	Codonopsis
30	Cola
31	Convallaria
32	Corni fructus
33	Cornus officinalis
34	Corydalis
35	Cowslip
36	Cramp bark
37	Crocetin
38	Crocin
39	Crocus
40	*Crocus sativus* L.
41	Cyclothymia
42	Cypripedium
43	Damiana
44	Datura
45	Deadly nightshade
46	Dioscorea
47	Dong quai
48	Echium amoenum
49	Eleutherococcus
50	Ephedra sinica
51	Ephedrae herba
52	*Eschscholzia*
53	Ferula
54	Ganoderma
55	Gelsemium
56	Ginseng
57	Glycyrrhiza
58	Goldenrod
59	Gotu kola
60	Gou qi zi
61	Green tea
62	Guarana
63	Hange-koboku-to
64	Happiness bark
65	Henbane
66	Herbal medicine
67	Herbs
68	Hoelen
69	Hops
70	Humulus
71	Hyoscyamus
72	Hypericum
73	Indian snakeroot
74	Jamaican dogwood
75	Jimson weed
76	Kampo
77	Kampo Medicine
78	Kanpo
79	Kanpo Medicine
80	Kava
81	Kava-Kava
82	Kola nut
83	korean ginseng
84	Koso-San
85	Lactuca
86	Lady's slipper
87	Lavandula
88	Lavender
89	Lemon balm
90	Leonurus
91	Licorice
92	Lily
93	Linden
94	Lobelia
95	Lotus
96	Lycii Fructus
97	Lycium barbarum
98	Ma-Huang
99	Magnolia
100	Marrubium
101	Matricaria
102	Melissa
103	Mentha
104	Mistletoe
105	Mitchella
106	Motherwort
107	Mystica
108	Nelumbo
109	Nepeta
110	Nervine
111	Nutmeg
112	Nux vomica
113	Oat
114	Ocimum
115	Opium poppy
116	Panax
117	Papaver
118	Passiflora
119	Passion flower
120	Paullinia
121	Peppermint
122	Phytomedicine
123	Phytotherapy
124	Pinellia ternate
125	Pinelliae rhizome
126	Piper
127	Piper methysticum
128	Piscidia
129	Plant extract
130	Plant preparation
131	Poria
132	Primula
133	Prunus
134	Pulsatilla
135	Rauvolfia
136	Rauwolfia
137	Rehmannia
138	Reishi
139	Rhodiola
140	Rhodiola rosea
141	Rosemary
142	Rosmarinus
143	Rosmarinus
144	Saffron
145	Safranal
146	Saffron extract
147	Saint John's wort
148	Sceletium
149	Schisandra
150	Scutellaria
151	Shan zhu
152	Sheng ban xia
153	Siberian ginseng
154	skullcap
155	Solidago
156	Stachys
157	St John's Wort
158	Sutherlandia
159	Syzygium
160	Thornapple
161	Thymoleptic
162	Tilia
163	Traditional Chinese medicine
164	Tulsi
165	Turnera
166	Valerian
167	Valeriana
168	Verbena
169	Vervain
170	Viburnum
171	Viscum
172	White horehound
173	Withania
174	Wild cherry
175	Wild lettuce
176	Wild yam
177	Xiang Su San
178	Yan hu suo
179	Yarrow
180	Yellow jasmine
181	Zizyphus
182	Free AND Easy Wanderer Plus
1. Bipolar depression	
2. Bipolar disorder	
3. Bipolar Disorder	
4. Mania	
5. Manic disorder	
6. Manic state	
