# Supplementary material for: Mania Associated With Herbal Medicines, Other Than Cannabis: A Systematic Review and Quality Assessment of Case Reports
Source: Front Psychiatry. 2018 Jul 6;9:280. doi: 10.3389/fpsyt.2018.00280 (PMC6043668; doi:10.3389/fpsyt.2018.00280)
Supplement: Supplementary file 1 [file Table_1.pdf]

Table 1. Details of each case report

Author (date)	Gender (age years)	Name of preparation	Dose of preparation	Reason for taking HM	Time to onset of manic symptoms	Concurrent medications	Mental State at presentation	Diagnosis	Hospitalized	Psychiatric history	Family psychiatric history	Medical history	Treatment outcomes
Barbenel et al. (2000)	M (28)	St John's wort	Unknown	Depression	5-8 weeks	Sertraline 50mg daily	over-aroused, distractible, flight of ideas, grandiose delusions	Manic episode	Yes	Referred for assessment of symptoms of depression	Depression in 5 brothers	Concomitant testosterone since bilateral orchidectomy. Wolff-Parkinson-White	Unknown
Dalwood et al. (2015)	M (39)	St John's Wort	"twice recommended dosage"	Low mood	4 weeks	None	Manic symptoms not elaborated	Manic episode	Yes	None	None	Unknown	Mania resolved after cessation of St John's wort and commencement of mood stabilizer medications
Fahmi et al. (2002)	F (28)	Hypericum	Average 18g/day	Depressive symptoms	2 weeks	None	Hyperactive, disorganized, pressured speech. Mood elevated, irritable. Flight of ideas, paranoid delusions.	Acute mania	Yes	18-month history of depression	None	None	Olanzapine and sodium valproate (doses unknown). Manic symptoms gradually resolved over three to four weeks
Moses & Mallinger (2000)	F (70)	St John's wort	3 x 300 then 2 x 300 mg/day	Depression	2 weeks	Notriptyline 75mg, bupropion 75mg daily	Insomnia, early waking, reckless spending, increased activity, careless	Not listed	Unclear	8 year history of recurrent depression	None	Ménière's disease, left internal capsule infarct	After two weeks she reported sleeping 8hrs per night, good spirits, less careless, not over-spending. Decreased St John's wort to one tablet, added valproic acid 750mg per day
Moses & Mallinger (2000)	M (53)	St John's wort	900 mg/day	Depression	Shortly after	Unknown	increased self-esteem, racing thoughts, talkative, sleep decreased, libido increased, reckless spending, driving faster, visual hallucinations.	Current diagnosis not listed	Unclear	Behaviors consistent with bipolar type II	Son bipolar type I, daughter depression	None	Discontinued St John's wort initiated lithium 900mg per day
Moses & Mallinger (2000)	F (61)	St John's wort	Unknown	Improve energy	2 weeks	Olanzepine 20mg daily, lithium	Flamboyantly dressed, referential delusions, paranoia, erotomanic	Psychotic Mania	Yes	Bipolar type I disorder	Unknown	hysterectomy, benign pulmonary nodules, thyromegaly	Re-admission after 3 days for suspected medication-related delirium
Nierenberg, Burt et al. (1999)	M (20)	St John's wort	0.2 % hypericum, 300 mg tds	Major depression	3 days	None	Extreme agitation, irritability, pressured speech, pacing, anxiety	Not listed	Yes	Depression	Uncle bipolar disorder	None	Discontinued St John's wort. Lithium 450mg twice day, clonazepam
Nierenberg, Burt et al. (1999)	F (51)	St John's wort	300 mg tds	Stressed and depressed	A few days	None	Speech disorganized, giggling uncontrollably, hypermotoric, hypersexual.	Not listed	Yes	Psychotic mania	Unknown	None	Lithium 600mg bd
O'Breasail & Argouarch (1998)	M (76)	St John's wort	Unknown	Depression	6 weeks	Venlafaxine 37.5 mg tds	Increased drive, overactive, pressured speech, irritability, euphoric grandiose plans. Reduced sleep and concentration	Hypomania	Yes	Depression	Depression & suicide	Cerebral vascular accident affecting right side. Atrial flutter	Valproate
O'Breasail & Argouarch (1998)	M (28)	St John's wort	Unknown	Posttraumatic stress symptoms	Approximately 3 months	None	Flight of ideas, pressured speech, agitated, irritable, grandiose delusions	Bipolar disorder, acute manic episode	Yes	Posttraumatic stress symptoms	Mother depression	Shoulder injury	Mood stabilization with lithium carbonate
Raja & Azzoni (2006)	F (47)	Hypericum, Ilex paraguayensis, cannabis.	Undetermined dosage	Major depression	6 months	Previously paroxetine 20mg daily	Mixed state, unstable mood, crying, dysphoric, irritable, logorrhea, motor hyperactivity, distractability, agitation, excessive sexual arousal, insomnia	Mixed state	Unclear	Major depression	Not listed	Not listed	Oxcarbazepine (up to 1200mg), clonazepam (up to 4mg) per day
Raja & Azzoni (2006)	M (32)	Hypericum	Dosage unknown	Depression	4 weeks	None	Sad, anxious, guilt, nervous, irritable, inconsistently hyperactive with racing thoughts, logorrhea, distractable, decreased sleep	Bipolar disorder, mixed state	Yes	Depression	Cousin schizoaffective, bipolar type	Not listed	Full remission after 11 days, discharged on valproate
Schneck (1998)	F (47)	St John's wort	0.1% tincture	Depression	10 days	Previously sertraline 50 mg daily	Racing and distorted thoughts, increased irritability, hostility, aggressive behavior, decreased sleep.	Hypomania	Unclear	Panic disorder and major depressive episode	Unknown	Not listed	Discontinued St John's wort, complete resolution of symptoms in 2 days
Spinella & Eaton (2002)	F (42)	Ginkgo biloba, melatonin, St John's wort	Unspecified doses	Depression	Past several weeks	Fluoxetine, buspirone	Disrupted sleep-wake cycle, insomnia, racing thoughts, agitation, pressured speech.	Hypomania	Unclear	Depression and anxiety	Unknown	Cervical sprain, concussion	Advised to cease all non-prescription medication, remained depressed
Engelberg et al. (2001)	M (26)	Chinese red ginseng root	250mg 2-3 caps per day	Boost energy	2 months	None	Pressured speech, racing thoughts, grandiosity, irritability. Thought form circumstantial, tangential, loose associations, poor insight.	Manic symptoms	Yes	None	None	None	Ceased the supplement 14 hours prior to admission. Valproic acid 500mg bd, Lorazepam 1mg daily. Euthymic after 10 days
Gonzalez-Seijo et al.(1995)	F (35)	Panax ginseng	"1 tablet" daily	Unknown	10 days	Previously lithium carbonate 1200mg, amitriptyline 75 mg daily	Euphoric, hyperactive, talkative, singing, spending and affective lability, irritable and aggressive. Little sleep	Manic episode	Yes	Depression with hospitalization	Unknown	None	Haloperidol 7.5mg, lithium carbonate 1000 mg per day. Symptoms resolved in 48 hours
Norelli & Xu (2014)	M (23)	Asian red ginseng	Estimated 15g ginseng daily	Boost energy	1 month	None	Increased psychomotor activity, pacing, pressured speech, racing disorganized thoughts, anxious, irritable, labile affect, auditory hallucinations	Acute manic psychosis	Yes	None	None	None	Risperidone 1mg daily. Symptoms remitted after 3 days
Norelli & Xu (2014)	M (79)	Korean ginseng, yohimbine	Estimated 20g ginseng daily	Erectile dysfunction	2 months	Unknown	Motor restlessness, pressured speech, anxious mood, labile affect, racing disorganized thoughts, paranoid ideas	Acute manic psychosis	Unclear	Substance-induced hypomanic episode associated with yohimbine	Unknown	Mild hypertension	"a short course of antipsychotic treatment"
