# Supplementary material for: Mania Associated With Herbal Medicines, Other Than Cannabis: A Systematic Review and Quality Assessment of Case Reports
Source: Front Psychiatry. 2018 Jul 6;9:280. doi: 10.3389/fpsyt.2018.00280 (PMC6043668; doi:10.3389/fpsyt.2018.00280)
Supplement: Supplementary file 2 [file Table_2.pdf]

Table 2. The quality of individual case reports

| Publication | Case Report Quality Assessment Score | Validated instrument to assess causality | Botanical name of HM | Herbal Material assayed for authentication | HM composition detailed* | Brand name and manufacturer stated | HM dosage specified |
|---|---|---|---|---|---|---|---|
| Barbenel et al 2000 | 26 upper medium | No | Yes | No | No | No | No |
| Dalwood et al 2015 | 23 upper medium | No | Yes | No | No | No | No |
| Fahmi et al 2002 | 29 high | No | Yes | No | No | No | Yes |
| Moses & Mallinger 2000 | 30 high | No | Yes | No | No | No | Yes, Yes, No |
| Nierenberg et al 1999 | 34 high | No | Yes | No | No | No | Yes, Yes |
| O'Breasail & Argouarch 1998 | 19 lower medium | No | No | No | No | No | No, No |
| Raja & Azzoni 2004 | 21 lower medium | No | Yes | No | No | No | No, No |
| Schneck 1998 | 26 upper medium | No | Yes | No | No | No | Yes |
| Spinella & Eaton 2002 | 27 upper medium | No | Yes | No | No | No | No |
| Engelberg et al 2001 | 33 high | No | Yes | No | No | No | Yes |
| Gonzalez-Seijo et al 1995 | 21 lower medium | No | Yes | No | No | No | No |
| Norelli & Xu 2015 | 27 upper medium | No | Yes | No | No | No | Yes, Yes |
| Vazquez & Aguera-Ortiz 2012 | 27 upper medium | No | Yes | No | Yes | No | Yes |
| Cotovio & Oliveira-Maia 2017 | 23 upper medium | No | Yes | No | No | No | No |
| Hendrickson et al 2016 | 18 lower medium | No | Yes | No | No | No | Yes, Yes, Yes |
| Boerth & Caley 2003 | 21 low medium | Yes | Yes | No | No | No | No |
| Capwell 1995 | 30 high | No | Yes | No | No | No | No |
| Emmanuel et al 1998 | 18 lower medium | No | No | No | No | No | No |
| Guzel Ozdemir et al 2015 | 15 lower medium | No | No | No | No | Yes | Yes |
| Katz 2000 | 30 high | No | No | No | No | Yes | Yes |
| Chong 2000 | 26 upper medium | No | No | Yes | No | No | No, No |
| Partin & Pushkin 2004 | 23 upper medium | No | Yes | No | No | No | Yes |
| Narasimha et al 2013 | 28 upper medium | No | No | No | No | Yes | Yes |
| Saatcioglu et al 2007 | 16 lower medium | No | No | No | Yes | No | No |
| Kelly et al 2001 | 22 upper medium | No | No | No | No | No | No |
| Khalid et al 2016 | 31 high | No | Yes | No | Yes | No | Yes |

*Note: *detailed both plant part used and extract type*
